# Supplementary material for: Hot spot prediction in protein-protein interactions by an ensemble system
Source: BMC Syst Biol. 2018 Dec 31;12(Suppl 9):132. doi: 10.1186/s12918-018-0665-8 (PMC6311905; doi:10.1186/s12918-018-0665-8)
Supplement: Supplementary file 2 — All relevant MATLAB code. A simple Matlab implement of our predictor. (ZIP 19,742 kb) [file 12918_2018_665_MOESM2_ESM.zip › Additional file 2/Read ASA/ASA website/dbMPIKT/1MLCA.html]

 NetSurfP - Protein Surface Accessibility and Secondary Structure Predictions

  

|  |  |  |
| --- | --- | --- |
|  |  | NetSurfP - Protein Surface Accessibility and Secondary Structure Predictions  Technical University of Denmark |

  


---

```
# For publication of results, please cite:
# A generic method for assignment of reliability scores applied to solvent accessibility predictions.
# Bent Petersen, Thomas Nordahl Petersen, Pernille Andersen, Morten Nielsen and Claus Lundegaard
# BMC Structural Biology 2009, 9:51 doi:10.1186/1472-6807-9-51
#
# Column 1: Class assignment - B for buried or E for Exposed - Threshold: 25% exposure, but not based on RSA
# Column 2: Amino acid
# Column 3: Sequence name
# Column 4: Amino acid number
# Column 5: Relative Surface Accessibility - RSA
# Column 6: Absolute Surface Accessibility
# Column 7: Z-fit score for RSA prediction
# Column 8: Probability for Alpha-Helix
# Column 9: Probability for Beta-strand
# Column 10: Probability for Coil
E D  1MLCA                  1    0.817 117.686   0.972   0.003   0.003   0.994
B I  1MLCA                  2    0.224  41.440  -0.549   0.003   0.718   0.279
E E  1MLCA                  3    0.466  81.340   1.523   0.001   0.900   0.099
B L  1MLCA                  4    0.123  22.540   0.629   0.001   0.900   0.099
E T  1MLCA                  5    0.450  62.429   0.680   0.002   0.816   0.182
B Q  1MLCA                  6    0.147  26.218   0.565   0.004   0.514   0.481
E S  1MLCA                  7    0.351  41.125   0.139   0.004   0.138   0.858
E P  1MLCA                  8    0.496  70.340  -1.291   0.018   0.019   0.964
E A  1MLCA                  9    0.527  58.075  -1.277   0.018   0.088   0.893
E T  1MLCA                 10    0.363  50.334  -1.434   0.021   0.451   0.528
B L  1MLCA                 11    0.246  45.134  -0.287   0.021   0.756   0.223
E S  1MLCA                 12    0.488  57.240  -0.702   0.018   0.846   0.136
B V  1MLCA                 13    0.209  32.077   0.012   0.023   0.655   0.322
E T  1MLCA                 14    0.447  61.943   0.278   0.004   0.420   0.576
E P  1MLCA                 15    0.523  74.157  -0.018   0.004   0.085   0.910
E G  1MLCA                 16    0.674  53.052  -0.988   0.005   0.015   0.979
E D  1MLCA                 17    0.489  70.422   0.331   0.004   0.138   0.858
E S  1MLCA                 18    0.536  62.796   1.025   0.003   0.718   0.279
B V  1MLCA                 19    0.066  10.206   0.200   0.001   0.900   0.099
E S  1MLCA                 20    0.426  49.904   1.579   0.001   0.959   0.040
B L  1MLCA                 21    0.050   9.063   0.417   0.001   0.959   0.040
E S  1MLCA                 22    0.326  38.231   0.641   0.001   0.900   0.099
B C  1MLCA                 23    0.026   3.608   0.341   0.001   0.900   0.099
E R  1MLCA                 24    0.393  90.066   0.867   0.001   0.900   0.099
B A  1MLCA                 25    0.165  18.227  -1.042   0.002   0.816   0.182
E S  1MLCA                 26    0.372  43.587  -0.953   0.004   0.514   0.481
E Q  1MLCA                 27    0.589 105.178  -2.444   0.004   0.085   0.910
E S  1MLCA                 28    0.458  53.713  -2.384   0.005   0.045   0.951
E I  1MLCA                 29    0.486  89.873  -1.566   0.004   0.085   0.910
E S  1MLCA                 30    0.485  56.877  -2.166   0.004   0.085   0.910
B N  1MLCA                 31    0.300  43.861  -1.043   0.004   0.197   0.799
B N  1MLCA                 32    0.148  21.726   0.362   0.004   0.616   0.381
B L  1MLCA                 33    0.026   4.706   0.558   0.002   0.816   0.182
B H  1MLCA                 34    0.074  13.388   0.452   0.001   0.900   0.099
B W  1MLCA                 35    0.026   6.253   0.872   0.006   0.962   0.032
B Y  1MLCA                 36    0.049  10.407   0.705   0.001   0.959   0.040
B Q  1MLCA                 37    0.140  25.040   0.807   0.001   0.900   0.099
B Q  1MLCA                 38    0.129  22.986   0.426   0.003   0.718   0.279
E K  1MLCA                 39    0.402  82.609   1.735   0.004   0.138   0.858
E S  1MLCA                 40    0.534  62.561  -0.447   0.005   0.015   0.979
E H  1MLCA                 41    0.721 131.095  -2.253   0.005   0.015   0.979
E E  1MLCA                 42    0.562  98.112   0.136   0.018   0.019   0.964
E S  1MLCA                 43    0.335  39.239  -0.521   0.018   0.047   0.935
B P  1MLCA                 44    0.142  20.207  -0.478   0.020   0.205   0.775
E R  1MLCA                 45    0.337  77.196   0.215   0.023   0.655   0.322
B L  1MLCA                 46    0.101  18.420  -0.039   0.002   0.816   0.182
B L  1MLCA                 47    0.039   7.123  -0.109   0.001   0.900   0.099
B I  1MLCA                 48    0.023   4.329   0.292   0.001   0.959   0.040
B K  1MLCA                 49    0.077  15.921  -0.150   0.001   0.959   0.040
B Y  1MLCA                 50    0.089  18.998  -0.288   0.001   0.900   0.099
B V  1MLCA                 51    0.112  17.230  -0.674   0.004   0.616   0.381
B S  1MLCA                 52    0.148  17.369  -0.871   0.021   0.279   0.699
E Q  1MLCA                 53    0.531  94.765  -1.421   0.018   0.088   0.893
E S  1MLCA                 54    0.431  50.537  -0.876   0.018   0.047   0.935
E S  1MLCA                 55    0.606  71.000  -2.269   0.018   0.019   0.964
E S  1MLCA                 56    0.686  80.341  -1.838   0.018   0.047   0.935
E G  1MLCA                 57    0.524  41.231  -2.021   0.018   0.047   0.935
E I  1MLCA                 58    0.410  75.850  -0.774   0.018   0.047   0.935
E P  1MLCA                 59    0.524  74.327  -1.441   0.005   0.045   0.951
E S  1MLCA                 60    0.559  65.515  -2.564   0.004   0.138   0.858
E R  1MLCA                 61    0.351  80.448  -0.741   0.004   0.420   0.576
B F  1MLCA                 62    0.174  34.902  -1.969   0.004   0.616   0.381
E S  1MLCA                 63    0.385  45.075  -1.298   0.003   0.718   0.279
E G  1MLCA                 64    0.348  27.411  -2.411   0.004   0.616   0.381
E S  1MLCA                 65    0.414  48.544  -1.707   0.004   0.514   0.481
E G  1MLCA                 66    0.437  34.384  -1.523   0.004   0.420   0.576
E S  1MLCA                 67    0.537  62.960  -2.076   0.004   0.197   0.799
E G  1MLCA                 68    0.538  42.380  -1.770   0.004   0.138   0.858
E T  1MLCA                 69    0.389  53.996  -1.430   0.005   0.262   0.733
E D  1MLCA                 70    0.376  54.239  -0.289   0.004   0.616   0.381
B F  1MLCA                 71    0.107  21.495  -0.600   0.002   0.816   0.182
B T  1MLCA                 72    0.229  31.790  -0.282   0.001   0.900   0.099
B L  1MLCA                 73    0.041   7.544  -0.821   0.001   0.900   0.099
E S  1MLCA                 74    0.269  31.480   0.547   0.001   0.900   0.099
B I  1MLCA                 75    0.062  11.433   0.409   0.001   0.900   0.099
E N  1MLCA                 76    0.333  48.707   0.418   0.021   0.756   0.223
E S  1MLCA                 77    0.353  41.360  -0.476   0.022   0.552   0.426
B V  1MLCA                 78    0.130  19.966  -0.228   0.021   0.279   0.699
E E  1MLCA                 79    0.403  70.317   0.300   0.021   0.279   0.699
E T  1MLCA                 80    0.375  51.985  -0.861   0.021   0.279   0.699
E E  1MLCA                 81    0.552  96.469  -1.065   0.019   0.141   0.840
E D  1MLCA                 82    0.555  79.976  -0.868   0.018   0.088   0.893
E F  1MLCA                 83    0.473  94.911  -1.318   0.004   0.138   0.858
E G  1MLCA                 84    0.509  40.090  -0.492   0.004   0.420   0.576
E M  1MLCA                 85    0.395  79.120   0.524   0.002   0.816   0.182
B Y  1MLCA                 86    0.111  23.806   1.168   0.002   0.816   0.182
B F  1MLCA                 87    0.124  24.907   0.557   0.002   0.816   0.182
B C  1MLCA                 88    0.079  11.035  -1.219   0.002   0.816   0.182
B Q  1MLCA                 89    0.099  17.717  -0.928   0.004   0.616   0.381
B Q  1MLCA                 90    0.148  26.379  -0.730   0.022   0.552   0.426
B S  1MLCA                 91    0.292  34.234  -1.125   0.021   0.451   0.528
E N  1MLCA                 92    0.358  52.397  -1.252   0.021   0.279   0.699
E S  1MLCA                 93    0.406  47.630  -1.667   0.020   0.205   0.775
E W  1MLCA                 94    0.341  82.035  -1.801   0.056   0.142   0.802
E P  1MLCA                 95    0.396  56.136  -1.208   0.020   0.205   0.775
E R  1MLCA                 96    0.382  87.455  -1.001   0.020   0.205   0.775
E T  1MLCA                 97    0.330  45.757  -1.639   0.020   0.205   0.775
B F  1MLCA                 98    0.191  38.294  -1.087   0.021   0.279   0.699
E G  1MLCA                 99    0.308  24.232  -1.510   0.022   0.359   0.619
E G  1MLCA                100    0.359  28.285  -1.008   0.021   0.279   0.699
E G  1MLCA                101    0.364  28.663  -1.201   0.021   0.279   0.699
E T  1MLCA                102    0.334  46.256  -1.249   0.021   0.279   0.699
E K  1MLCA                103    0.382  78.680  -0.679   0.022   0.359   0.619
E L  1MLCA                104    0.291  53.355  -0.886   0.066   0.296   0.638
E E  1MLCA                105    0.319  55.782  -0.773   0.066   0.296   0.638
E I  1MLCA                106    0.354  65.509  -1.194   0.066   0.296   0.638
E K  1MLCA                107    0.402  82.609  -1.031   0.066   0.296   0.638
E R  1MLCA                108    0.418  95.653  -1.242   0.064   0.216   0.721
E A  1MLCA                109    0.483  53.183  -1.447   0.056   0.142   0.802
E D  1MLCA                110    0.474  68.275  -1.101   0.052   0.084   0.864
E A  1MLCA                111    0.405  44.598  -1.117   0.056   0.142   0.802
E A  1MLCA                112    0.386  42.526  -0.746   0.118   0.150   0.732
E P  1MLCA                113    0.413  58.661  -1.231   0.118   0.150   0.732
E T  1MLCA                114    0.395  54.856  -1.687   0.118   0.150   0.732
E V  1MLCA                115    0.326  50.168  -1.200   0.118   0.150   0.732
E S  1MLCA                116    0.396  46.470  -1.246   0.125   0.227   0.648
E I  1MLCA                117    0.433  80.179  -1.249   0.125   0.227   0.648
E F  1MLCA                118    0.385  77.330  -1.960   0.118   0.150   0.732
E P  1MLCA                119    0.359  50.900  -0.862   0.118   0.150   0.732
E P  1MLCA                120    0.434  61.599  -1.475   0.125   0.227   0.648
E S  1MLCA                121    0.368  43.094  -0.841   0.135   0.317   0.548
E S  1MLCA                122    0.366  42.919  -1.441   0.066   0.296   0.638
E E  1MLCA                123    0.361  63.102  -1.461   0.069   0.386   0.545
E Q  1MLCA                124    0.370  66.136  -1.658   0.066   0.296   0.638
E L  1MLCA                125    0.426  77.982  -0.926   0.066   0.296   0.638
E T  1MLCA                126    0.383  53.191  -1.117   0.020   0.205   0.775
E S  1MLCA                127    0.530  62.069  -1.635   0.019   0.141   0.840
E G  1MLCA                128    0.602  47.401  -1.812   0.004   0.138   0.858
E G  1MLCA                129    0.358  28.182  -0.194   0.005   0.336   0.660
B A  1MLCA                130    0.055   6.105  -0.699   0.004   0.616   0.381
B S  1MLCA                131    0.114  13.337  -0.022   0.001   0.900   0.099
B V  1MLCA                132    0.043   6.624   0.360   0.001   0.959   0.040
B V  1MLCA                133    0.065  10.037   0.011   0.001   0.959   0.040
B C  1MLCA                134    0.024   3.370   0.210   0.001   0.900   0.099
B F  1MLCA                135    0.146  29.222  -0.884   0.001   0.900   0.099
B L  1MLCA                136    0.086  15.747  -1.552   0.003   0.718   0.279
E N  1MLCA                137    0.308  45.091  -1.269   0.005   0.336   0.660
E N  1MLCA                138    0.494  72.366  -2.309   0.004   0.197   0.799
E F  1MLCA                139    0.510 102.277  -1.670   0.004   0.138   0.858
E Y  1MLCA                140    0.345  73.812  -1.804   0.004   0.138   0.858
E P  1MLCA                141    0.399  56.632  -1.526   0.004   0.197   0.799
E K  1MLCA                142    0.497 102.233  -1.909   0.005   0.336   0.660
E D  1MLCA                143    0.297  42.783   0.052   0.004   0.616   0.381
B I  1MLCA                144    0.043   7.863  -0.140   0.002   0.816   0.182
B N  1MLCA                145    0.195  28.533   0.481   0.001   0.900   0.099
B V  1MLCA                146    0.043   6.563   0.236   0.001   0.900   0.099
B K  1MLCA                147    0.142  29.168   0.199   0.006   0.962   0.032
B W  1MLCA                148    0.067  16.089  -0.151   0.011   0.918   0.071
E K  1MLCA                149    0.283  58.151   0.479   0.002   0.816   0.182
E I  1MLCA                150    0.319  58.997   0.902   0.004   0.514   0.481
E D  1MLCA                151    0.711 102.513   0.059   0.005   0.015   0.979
E G  1MLCA                152    0.760  59.836  -2.086   0.005   0.015   0.979
E S  1MLCA                153    0.661  77.504  -0.338   0.004   0.197   0.799
E E  1MLCA                154    0.549  95.928  -0.984   0.004   0.197   0.799
E R  1MLCA                155    0.316  72.433  -0.656   0.004   0.197   0.799
E Q  1MLCA                156    0.474  84.692  -0.910   0.005   0.336   0.660
E N  1MLCA                157    0.369  53.963  -1.130   0.004   0.420   0.576
B G  1MLCA                158    0.275  21.619  -1.693   0.004   0.616   0.381
B V  1MLCA                159    0.259  39.839  -1.092   0.002   0.816   0.182
B L  1MLCA                160    0.239  43.798  -0.644   0.001   0.900   0.099
E N  1MLCA                161    0.400  58.545  -1.017   0.021   0.756   0.223
B S  1MLCA                162    0.235  27.554  -1.580   0.022   0.552   0.426
E W  1MLCA                163    0.332  79.942  -0.427   0.021   0.451   0.528
E T  1MLCA                164    0.461  63.913  -0.891   0.021   0.279   0.699
E D  1MLCA                165    0.389  56.113  -0.860   0.019   0.141   0.840
E Q  1MLCA                166    0.548  97.891  -1.718   0.019   0.141   0.840
E D  1MLCA                167    0.612  88.189  -2.063   0.018   0.088   0.893
E S  1MLCA                168    0.596  69.863  -1.542   0.018   0.047   0.935
E K  1MLCA                169    0.516 106.162  -0.604   0.018   0.019   0.964
E D  1MLCA                170    0.674  97.066  -1.788   0.005   0.015   0.979
E S  1MLCA                171    0.541  63.464  -1.975   0.005   0.045   0.951
E T  1MLCA                172    0.353  48.933   0.208   0.004   0.420   0.576
B Y  1MLCA                173    0.079  16.882  -1.000   0.004   0.616   0.381
B S  1MLCA                174    0.117  13.724  -1.166   0.002   0.816   0.182
B M  1MLCA                175    0.068  13.507  -1.381   0.001   0.900   0.099
B S  1MLCA                176    0.144  16.830  -0.343   0.001   0.959   0.040
B S  1MLCA                177    0.041   4.770  -1.557   0.001   0.959   0.040
B T  1MLCA                178    0.192  26.617  -0.106   0.001   0.959   0.040
B L  1MLCA                179    0.040   7.287  -0.125   0.001   0.900   0.099
E T  1MLCA                180    0.305  42.248   0.793   0.001   0.900   0.099
B L  1MLCA                181    0.069  12.616   0.476   0.002   0.816   0.182
E T  1MLCA                182    0.357  49.516   1.045   0.021   0.451   0.528
E K  1MLCA                183    0.449  92.256  -0.878   0.021   0.279   0.699
E D  1MLCA                184    0.521  75.134  -1.111   0.020   0.205   0.775
E E  1MLCA                185    0.488  85.254   0.282   0.019   0.141   0.840
B Y  1MLCA                186    0.247  52.784  -0.932   0.018   0.088   0.893
E E  1MLCA                187    0.662 115.669   0.276   0.005   0.045   0.951
E R  1MLCA                188    0.618 141.591   0.555   0.005   0.045   0.951
E H  1MLCA                189    0.603 109.777  -0.597   0.004   0.085   0.910
E N  1MLCA                190    0.447  65.397  -0.056   0.005   0.336   0.660
E S  1MLCA                191    0.435  50.982   1.575   0.002   0.816   0.182
B Y  1MLCA                192    0.061  13.100   1.044   0.001   0.959   0.040
B T  1MLCA                193    0.273  37.796   1.791   0.001   0.959   0.040
B C  1MLCA                194    0.022   3.047   0.931   0.001   0.959   0.040
B E  1MLCA                195    0.205  35.831   1.202   0.001   0.959   0.040
B A  1MLCA                196    0.074   8.133  -0.056   0.001   0.959   0.040
E T  1MLCA                197    0.363  50.320   0.565   0.001   0.959   0.040
B H  1MLCA                198    0.135  24.484   0.397   0.002   0.816   0.182
E K  1MLCA                199    0.608 125.127  -0.096   0.005   0.262   0.733
E T  1MLCA                200    0.630  87.381  -1.926   0.005   0.045   0.951
E S  1MLCA                201    0.439  51.498  -0.886   0.005   0.045   0.951
E T  1MLCA                202    0.594  82.416  -0.054   0.005   0.045   0.951
E S  1MLCA                203    0.537  62.878   0.352   0.019   0.141   0.840
E P  1MLCA                204    0.398  56.490   0.272   0.005   0.336   0.660
B I  1MLCA                205    0.151  27.880  -0.686   0.003   0.718   0.279
E V  1MLCA                206    0.539  82.814   0.425   0.001   0.900   0.099
E K  1MLCA                207    0.325  66.791   1.260   0.001   0.900   0.099
E S  1MLCA                208    0.494  57.838   0.120   0.002   0.816   0.182
B F  1MLCA                209    0.094  18.786   0.617   0.003   0.718   0.279
E N  1MLCA                210    0.529  77.504   1.877   0.004   0.514   0.481
E R  1MLCA                211    0.452 103.577   0.599   0.019   0.141   0.840
E N  1MLCA                212    0.660  96.697  -0.915   0.018   0.088   0.893
E E  1MLCA                213    0.637 111.319   1.525   0.018   0.088   0.893
E C  1MLCA                214    0.441  61.874  -1.260   0.003   0.003   0.994

Explain the output.  Go back.
```

---
